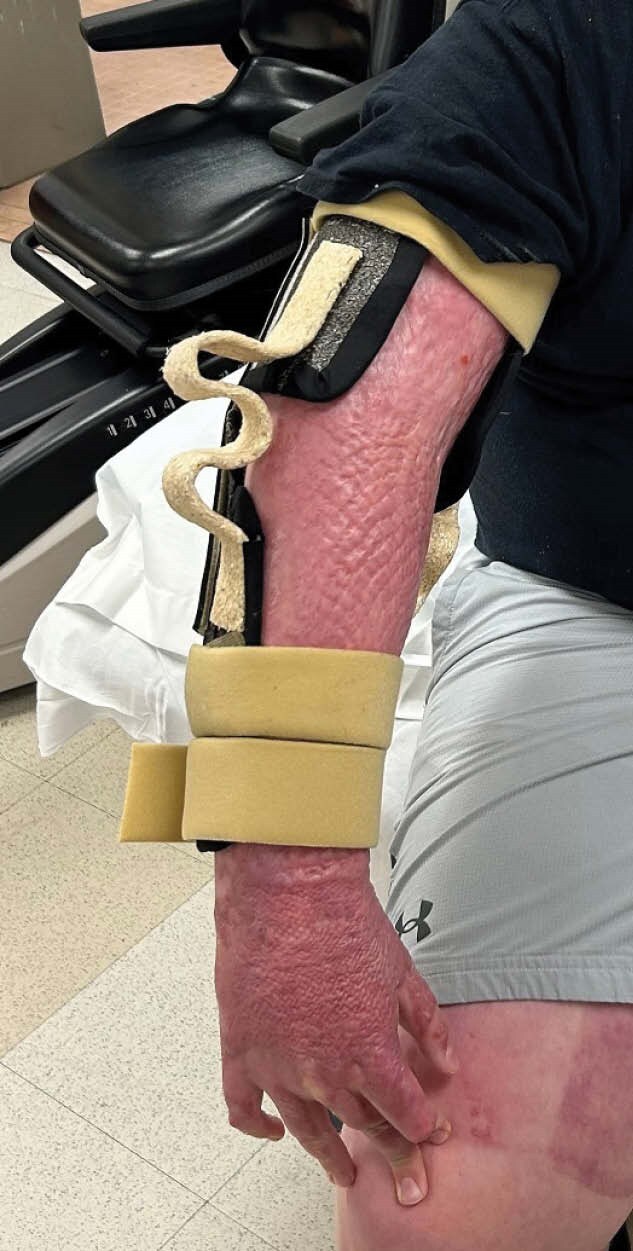# 1008 Utilization of Serial Casting and Dynamic Functional Splint to Manage Burn Scar Elbow Flexion Contractures

**DOI:** 10.1093/jbcr/iraf019.539

**Published:** 2025-04-01

**Authors:** Joshua Rodriguez, Joshua Rodriguez, Scott Vocke, Brooke Dean

**Affiliations:** Johns Hopkins Bayview Medical Center; Johns Hopkins Bayview Medical Center; Johns Hopkins Bayview Medical Center; Johns Hopkins Bayview Medical Center

## Abstract

**Introduction:**

Burn scar contracture (BSC) is a complication that can occur during the remodeling phase of wound healing. Serial casting is an effective intervention used to progressively increase ROM of joints impacted from BSC by providing low load, long duration stress. However, there are several disadvantages to immobilizing joints with casts such as, development of disuse atrophy and limiting functional use of a casted extremity. Even after ROM gains are made, immature scar continues to be suspectable to re-contracture. Dynamic splints can offer several benefits that serial casting cannot including allowing for functional movement and splint donning/doffing. This case series investigates the utilization of serial casting followed by the use of a novel dynamic functional elbow extension splint to maintain and improve on ROM gains from serial casting.

**Methods:**

2 patients with elbow flexion BSC were included in this case series and underwent initial serial casting using fiberglass casting tape. Subject A presented with bilateral elbow BSC and serial casting was performed requiring 1 cast over a 2-days. Subject B presented with a right elbow BSC and serial casting was performed requiring 2 casts over 6-days. Subsequently, dynamic elbow extension splints were fabricated using a novel moldable wood-composite and a biodegradable polymer splinting material (See image). Splint wearing schedules were prescribed and ROM measurements were tracked at each subsequent visit.

**Results:**

Significant elbow extension PROM improvements were made from serial casting in both subjects: Subject A – Right Elbow (RE): 30°; Left Elbow (LE): 46°. Subject B – RE: 55°. Further PROM elbow extension gains were made with initiation of dynamic splinting and re-contracture prevented: Subject A - RE: 5°; LE: 7°. Subject B – RE: 10°.

**Conclusions:**

Utilizing serial casting for initial treatment of elbow flexion BSC in these 2 subjects was effective in achieving significant extension PROM improvements. Follow-up use of a novel dynamic functional elbow extension splint was successful in maintaining these ROM gains and further improved each of the contractures.

**Applicability of Research to Practice:**

Our results supported the effectiveness of serial casting in treating BSC. However, there is continued risk for rebound contracture during the scar maturation process. In our limited case series, we were able to prevent re-contracture of all 3 elbow contractures investigated and continue to make ROM improvements using a dynamic elbow extension splint. In addition, this splint allows patients to participate in functional activities requiring elbow AROM while still providing scar with a low load dynamic stretch. We hope that this case series will aide other burn rehabilitation clinician in managing elbow flexion contractures caused by BSC.

**Funding for the Study:**

N/A